# Modulation of immune responses in stress by Yoga

**DOI:** 10.4103/0973-6131.43541

**Published:** 2008

**Authors:** Sarika Arora, Jayashree Bhattacharjee

**Affiliations:** Department of Biochemistry, GB Pant Hospital, New Delhi - 110 002, India; 1 Department of Biochemistry, Lady Hardinge Medical College, New Delhi - 110 002, India

**Keywords:** Hypothalamo-pituitary adrenal axis, immune reponse, neuroendocrine factors, stress, Yoga

## Abstract

Stress is a constant factor in today's fastpaced life that can jeopardize our health if left unchecked. It is only in the last half century that the role of stress in every ailment from the common cold to AIDS has been emphasized, and the mechanisms involved in this process have been studied. Stress influences the immune response presumably through the activation of the hypothalamic-pituitary adrenal axis, hypothalamic pituitary-gonadal axis, and the sympathetic-adrenal-medullary system. Various neurotransmitters, neuropeptides, hormones, and cytokines mediate these complex bidirectional interactions between the central nervous system (CNS) and the immune system. The effects of stress on the immune responses result in alterations in the number of immune cells and cytokine dysregulation. Various stress management strategies such as meditation, yoga, hypnosis, and muscle relaxation have been shown to reduce the psychological and physiological effects of stress in cancers and HIV infection. This review aims to discuss the effect of stress on the immune system and examine how relaxation techniques such as Yoga and meditation could regulate the cytokine levels and hence, the immune responses during stress.

## INTRODUCTION

Stress is a common condition—a response to a physical threat or psychological distress that generates a host of chemical and hormonal reactions in the body. In mammals, these responses include changes that increase the delivery of oxygen and glucose to the heart and large skeletal muscles. The result of such a response to stress is physiological support for adaptive behaviors such as “fight or flight.” As a part of the adaptive response to stress, various body systems such as the autonomic, cardiovascular, gastrointestinal, and immune systems may be affected.[1]

Elliot and Eisdorfer's taxonomy distinguishes stressors into five categories based on two important dimensions: duration and course (*e.g*., discrete *vs* continuous).[2]

*(i) Acute time-limited stressors* involve challenges such as public speaking or mental arithmetic. (ii) *Brief naturalistic stressors*, such as academic examinations, are short-term challenges that occur during certain points in a person's life. (iii) In *stressful event sequences,* a focal life-changing event, such as the loss of a spouse or a major natural disaster, gives rise to a series of related challenges. Although affected individuals usually do not know exactly when these challenges will subside, they have a clear sense that at some point in the future they will. (iv) *Chronic stressors* usually pervade a person's life, forcing him or her to restructure his or her identity or social roles. These include stress induced by an extreme change in lifestyle, such as becoming permanently disabled from an accident, or a refugee forced out of one's native country by war, in which case there seems to be no light at the end of the tunnel. (v) *Distant stressors* are traumatic experiences that occurred in the distant past yet have the potential to continue influencing the body systems because of their long-lasting cognitive and emotional sequelae. Examples of distant stressors include child abuse or posttraumatic stress experienced by war veterans.[3]

The immune system is a network of glands, nodes, and organs that work to protect the body from bacteria, viruses, fungi, and other harmful organisms. The immune system requires a constant supply of energy and nutrients to maintain optimal function and performance. Toxins in the environment and in our food, poor diet, lack of or excessive exercise, and stress can all adversely affect the function of the immune system and can cause a decline in its proper activity. Without the immune system functioning at optimal levels, the body becomes subject to health problems.

Solomon first demonstrated the influence of stress on immune response in animals and human beings.[4] His findings showed that stress and central nervous system (CNS) lesions affect thymus-derived lymphocytes (T cells) and play a role in cell-cell interaction and the release of mediators from reacting lymphocytes. Stress-induced immune dysregulation has been shown to be significant enough to result in health consequences, including the reduction of the immune response to vaccines, delayed wound healing,[5,6] reactivation of latent herpes viruses such as Epstein–Barr virus (EBV)[7] as well as the enhancement of risk for more severe infectious disease.[8]

## BIDIRECTIONAL COMMUNICATION BETWEEN STRESS AND IMMUNE RESPONSES

**Figure 1:**
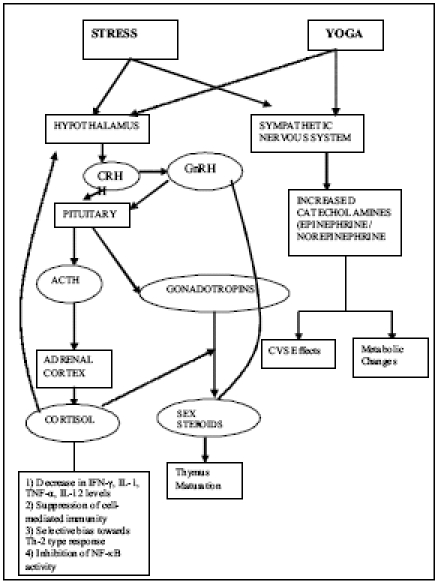
Neuroendocrine changes and the resultant immune responses associated with stress and the possible mechanism of action of Yoga. Solid lines indicate a stimulatory effect and dotted lines indicate an inhibitory effect. CRH: Corticotropin-Releasing Hormone, GnRH: Gonadotropin-releasing Hormone, ACTH: Adrenocorticotrophic Hormone, CVS: Cardiovascular system, IFN: Interferon, IL: Interleukin, TNF: Tumor Necrosis Factor, Th: T-helper, NF: Nuclear Factor

How does stress “get inside the body” to affect the immune response? The immune system is a network of glands, nodes, and organs that work to protect the body from bacteria, viruses, fungi, and other harmful organisms. The immune system requires a constant supply of energy and nutrients to maintain optimal function and performance. Toxins in the environment and in our food, poor diet, lack of or excessive exercise, and stress can all adversely affect the function of the immune system and can cause a decline in its proper activity. The effect of stress on immune system is mediated by a complex network of signals that function in a bidirectional manner in the nervous, endocrine, and immune systems [Figure 1]. The mediators of these interactions are mainly neurotransmitters, neuropeptides, hormones, and cytokines.

(i) The nervous system to immune system network involves sympathetic fibers descending from the brain into both primary (bone marrow and thymus) and secondary (spleen and lymph nodes) lymphoid tissues.[9] These fibers release a wide variety of substances that influence immune responses by binding to receptors on white blood cells.[9–11] Although all lymphocytes have adrenergic receptors, the differential density and sensitivity of adrenergic receptors on lymphocytes may affect their responsiveness to stress. For example, natural killer cells have both high-density and high-affinity β_2_ -adrenergic receptors whereas B cells have high density but lower affinity, and T cells have the lowest density.[12,13]
(ii) The endocrine-immune system network involves the hypothalamic–pituitary–adrenal axis (HPA), the sympathetic–adrenal–medullary axis (SAM), and the hypothalamic–pituitary–gonadal (HPG) axis. These endocrine axes secrete hormones that bind to specific receptors on white blood cells and have diverse regulatory effects on their distribution and function.[14] At the molecular level, human immune function is mediated by the release of cytokines from a variety of cells from the immune system and from endothelial cells. These cytokines stimulate the cellular release of specific compounds involved in the inflammatory response. The hormonal alterations induced by stress are responsible for the changes in the cytokine concentrations [Table 1].[15]

**Table 1:** Effect of hormones on Immune Response / cytokines

Corticotropin-releasing hormone	Activates Macrophages, inhibits IL-1 and IL-6 production
Adrenocorticotrophic hormone	Inhibits IFN-γ and IgG production and blocks macrophage activation by IFN-γ. Amplifies proliferation of B cells.
Growth hormone	Activates macrophages and enhances H_2_ O _2_ production
Gonadotropin-releasing hormone	Increases IL-2 receptor (IL-2R) expression, T and B-cell proliferation and serum Ig
Prolactin	Increases T-cell proliferation, IFN-γ, IL-2R, and macrophage function
Thyroid-stimulating hormone	Increases IL-2, GM-CSF, and Ig production
Luteinizing hormone	Enhances Il-2 stimulated T-cell production
Oxytocin and vasopressin	Increase IFN-γ production
Cortisol	Inhibits IFN-γ, IL-2, IL-6, and TNF-α; enhances IL-4 and TGF-β production; enhances immune cell expression of IL-1, IL-2, IL-6, and IFN-γ receptors
Estrogen	Increases T–cell proliferation and activity of the IFN-γ. gene promoter
Progesterone	Increases IL-4 production and CD30 expression
Adrenaline	Inhibits IL-1 and IL-2 production
Testosterone	Increases IL-10 production
Thyroxine	Activates T cells

IFN: Interferon, IgG: Immunoglobulin G, TGF: Transforming Growth Factor, IL: Interleukin, IL-2R: Interleukin -2 receptor, TNF: Tumor Necrosis Factor, GM-CSF: Granulocyte Monocyte Colony Stimulating Factor
(iii) Efforts to manage stressful events often lead to alcohol abuse or changes in sleeping patterns that could modify immune system processes.[16] Immunological activation in mammals results in a syndrome called *sickness behavior*, which consists of behavioral changes such as a reduction in activity, social interaction, sexual activity, as well as increased responsiveness to pain, anorexia, and depressed mood. This syndrome is probably adaptive as it results in energy conservation at a time when such energy is best directed towards fighting infection.[17]

## INFLUENCE OF HYPOTHALAMO-PITUITARY ADRENAL AXIS ON IMMUNE RESPONSE

The fine, homeostatic interactions between the hypothalamus (H) and the pituitary (P) and adrenal glands (A) constitute the HPA axis that controls reactions to stress and regulates various body processes including digestion, mood, sexuality, energy usage, and the immune system. The paraventricular nucleus of the hypothalamus synthesizes and secretes vasopressin and Corticotropin Releasing Hormone (CRH). These two peptides regulate the anterior lobe of the pituitary gland. CRH induces pro-opiomelanocortin (POMC) synthesis and ACTH secretion.[18] Adrenocorticotrophic Hormone (ACTH), in turn, stimulates the synthesis and secretion of adrenal steroid hormones (glucocorticoids), especially cortisol, which is a major stress hormone affecting many tissues in the body, including the brain. Cortisol acts on two types of receptors in the brain: mineralocorticoid and glucocorticoid receptors expressed by different types of neurons. Another important target of cortisol is the hippocampus, which is a major controlling center of the HPA axis. Cortisol acts on the hypothalamus and the pituitary gland in a negative feedback cycle to suppress CRH and ACTH production.[18]

HPA activation during inflammation is an important protective mechanism, as the resultant induction of endogenous corticosteroids restricts the immune reaction[19] and mediates lymphocyte compartmentalization. Cytokines including Interleukin (IL)-1, IL-6, leukemia inhibitory factor (LIF), and tumor necrosis factor (TNF) participate as mediators.[20] TNF-α levels have been found to be significantly lower in exam-taking students with high anxiety scores.[21]

### Effect of CRH and ACTH

Release of CRH from the hypothalamus is influenced by stress, by blood cortisol levels, and by the sleep/wake cycle. Besides its role in ACTH release, CRH occurs diffusely in the brain and serves as a neurotransmitter that mediates sympathetic arousal, providing an important link between the adrenocortical and autonomic branches of the stress response. Intracerebroventricular administration of CRH elevates plasma catecholamine concentrations, blood pressure, and the heart rate.[22] CRH also inhibits endotoxin-mediated production of IL-1 and IL-6 by human monocytes, and ACTH suppresses Interferon-gamma (IFN-γ) production by human lymphocytes.[23]

### Effect of Glucocorticoids

Glucocorticoid hormones being lipophilic, are capable of passing through the plasma membrane of all type of cells. They are capable of inhibiting cytokines; phospholipids, proteases, and oxygen metabolites, and hence, act as powerful immune regulators.[24] Glucocorticoid receptors present on macrophages and T-lymphocytes further mediate the immune regulation by glucocorticoids.[25] Glucocorticoids downregulate cytokine expression by binding and activating negative regulatory elements in the promoter region of cytokine genes.[26–28] Downregulated cytokines are IL-6, IL-2, IFN-γ,[23] IL-3, Granulocyte Monocyte Colony Stimulating Factor (GM-CSF), TNF-α, IL-4, and IL-8.[25]

Glucocorticoids tend to suppress cell-mediated immunity, but enhance immunoglobulin production.[29] Thus, an increase in IL-4 production is seen at all levels of glucocorticoids that inhibit IL-2 production.[30] Glucocorticoids tend to have a favorable bias towards the development of T cells that produce Th2 (T-helper2) cytokines.[31] The propensity of glucocorticoids to increase production of IL-4, IL-10, and transforming growth factor (TGF)-β, is consistent with their imparting a selective bias towards Th2 response.[32,33] Memory T cells are 100-fold less sensitive than naive T cells to inhibition by glucocorticoids,[32,33] raising the possibility that glucocorticoids have a greater role in regulating primary rather than secondary immune responses.

Glucocorticoids can also interfere with Nuclear Factor- Kappa B (NF-κB) activity[34,35] by transactivating its inhibitors. NF-κB regulates many cytokines produced by macrophages and Th cells.[36] Thus repression of NF-κB at a transcriptional level may inhibit the secretion of these cytokines.

Thus, glucocorticoids initially stimulate the immune system and then help to return it to baseline. It is only with major stressors of longer duration, or with major exposure to glucocorticoids, that the immune system does not just return to baseline, but plummets into immunosuppression.

## INFLUENCE OF HYPOTHALAMO-PITUITARY GONADAL (HPG) AXIS ON IMMUNE RESPONSE

The gonadotropin-releasing hormone (GnRH) and sex steroids play an important role in immune system modulation and development.

### Effect of GnRH

Immunoreactive and bioactive GnRH has been detected in human peripheral T cells (CD4+ and CD8+) and in a leukemic cell line (Jurkat) similar to T-lymphocytes.[37,38] The direct involvement of GnRH in thymus maturation and the development of cell-mediated and humoral immune responses in rats was first demonstrated by Morale *et al.* in 1991.[39] Zakharova *et al.* have confirmed the potential role of GnRH in prenatal and postnatal programming of immune cells in the rat embryo thymus.[40] In ageing male and female rats, the parallel decreases in thymic GnRH-binding sites and thymus weight is reversed by chronic (45 days) potent GnRH analogue (GnRH-*N* -ethylamide) treatment.[41] Interleukin-2 (IL-2) is an important cytokine in the proliferation and/or activation of T and B cells. IL-2 receptor (IL2R) expression is stimulated in rat thymocytes and splenocyte cultures incubated with native GnRH and its analogues in the absence of any other mitogenic stimulus, an effect that is reversed by GnRH antagonists.[42] Native GnRH significantly enhances *in vitro* IFN-γ production by human peripheral mononuclear cells.[43] In an animal model of immunodeficiency, total IgG levels and CD4+ lymphocytes increased after seven weeks of native GnRH administration, invoking a direct stimulatory effect of GnRH on immune function.[44] Sempowski *et al.*[45] have shown that the expression of IL-2, IL-10, and IL-14 decreases during thymic ageing.

### Effect of sex steroids

Experimental studies suggest that sex steroids influence immune cell development in primary lymphoid tissues (bone marrow and thymus) and in addition, have immunomodulatory effects on both peripheral T cell and B cell subsets in adult life.[46] 17 β-estradiol markedly increases the activity of the IFN-γ gene promoter in lymphoid cells[47] while progesterone induces transient IL-4 gene expression in established Th1 clones.[48] Estrogen receptor (ER) expression has been observed in thymocytes and thymic epithelial cells in both mice and humans.[49,50]

Experimental data have established that estrogen enhances the humoral immune response and may have an activating role in autoimmune disorders.[51] Androgen receptors (ARs) in turn, have been demonstrated in thymocytes,[52] but not in mature peripheral T and B lymphocytes.[53] Androgens exert considerable effects on the size and composition of the thymus. Early studies in rats and mice demonstrated that castration induced thymic enlargement, whereas testosterone replacement was associated with thymic regression, with a shift towards the expression of mature thymocytes. Mechanisms of androgen-induced thymus involution are incompletely understood, but may include decreased cell proliferation; changes in cell trafficking, and increased apoptosis. Olsen *et al.* suggested the role of apoptosis,[54] in which a single dose of testosterone in castrated mice markedly decreased thymic size within hours and increased DNA fragmentation. Testosterone specifically targets double-positive (CD8+CD4+) thymocytes for apoptosis by increasing TNF-α production.[55] A predominance is seen of the suppressor / cytotoxic CD4-CD8+ phenotype over the helper CD4+CD8-phenotype.[56] Although classical AR expression is absent in peripheral T cells, the net effect of androgen action (direct or indirect) seems to be an enhanced suppressor effect.[57]

## INTERACTION OF HPG AXIS WITH HPA AND SAM

Reproduction is inhibited by various components of the HPA/stress response. Interactions between CRH and the HPG axis are bidirectional. Glucocorticoids, POMC-like peptides, and CRH interfere with the stimulatory action of gonadotropins on sex steroid-producing cells, and may also decrease the responsiveness of the pituitary gland to GnRH.[58] In highly trained athletes and ballet dancers, the HPA axis remains chronically activated. Hence, high levels of cortisol and ACTH are observed along with low levels of Luteinizing Hormone (LH) in females and low testosterone concentrations in males.

Conversely, estrogen directly stimulates the CRH gene promoter and the central noradrenergic system. This may explain the preponderance of affective, anxiety, and eating disorders; mood cycles, and vulnerability to autoimmune and inflammatory disease, along with slightly higher cortisol levels in adult women.[59]

## INFLUENCE OF SAM AXIS ON IMMUNE RESPONSE

Noradrenergic sympathetic nerve fibers run from the CNS to both primary and secondary lymphoid organs[60] and neighboring immune cells.[61] In concert with the HPA axis, catecholamines modulate a variety of immune functions including cell proliferation, cytokine and antibody production, cytolytic activity, and cell trafficking.[11,61–63] In fact, activation of the HPA axis also results in secretion of catecholamines (epinephrine and norepinephrine) from the adrenal medulla.[63] In humans, approximately 80% of the catecholamine output of medulla is epinephrine.[64] Norepinephrine, on the other hand, is secreted by sympathetic nerve fibers in close proximity of the target tissues.

If acutely activated, these catecholaminergic systems can provide the body with a needed ‘boost’ to deal with an immediate threat. The typical and most obvious effect of stress-induced epinephrine and norepinephrine is the establishment of the primitive mammalian “fight or flight” reaction, in which there is an increased heart rate and increased blood flow to skeletal muscles. If the SAM is chronically activated, these molecules can dysregulate immune function.[60]

Exposure of ovarian cancer cell lines to increasing concentrations of norepinephrine or epinephrine have shown that both independently increased levels of phosphorylated Signal Transducers and activators of transcription (STAT)3 in a dose-dependent fashion. Immunolocalization and Enzyme Linked Immunosorbent Assay (ELISA) of nuclear extracts confirmed increased nuclear STAT3 in response to norepinephrine. Activation of STAT3 was inhibited by a blockade of the ß1- and ß2-adrenergic receptors with propranolol, and by blocking of protein kinase A with KT5720, but not with the α-receptor blockers—prazosin (α1) and/or yohimbine (α2). Catecholamine-mediated STAT3 activation was *not* inhibited by pretreatment with an anti-interleukin 6 (IL-6) antibody or with small interfering RNA (siRNA)-mediated decrease in IL-6 or gp130. The effects of STAT3 activation due to exposure to norepinephrine resulted in an increase in invasion and matrix metalloproteinase (MMP-2 and MMP-9) production, as well as invasion and *in vivo* tumor growth, which can be ameliorated by STAT3-targeting siRNA.[65,66].

## INFLUENCE OF OTHER HORMONES AND NEURO-ENDOCRINE FACTORS ON IMMUNE RESPONSE

Growth hormone (GH) activates human macrophages and primes monocytes for enhanced H_2_ O_2_ release.[67] When given to hypopituitary animals, GH augments antibody synthesis and skin graft rejection,[68] whereas prolactin (PRL) enhances macrophage function. PRL shares target transcription factors including interferon regulatory factor-1 (IRF-1) with IL-2. PRL receptors are expressed on T and Natural Killer (NK) cells and prolactin increases IL-2-stimulated NK-cell IFN-γ production.[69] Reduced prolactin release in response to bromocryptine administration is associated with a suppression of macrophage tumoricidal activity, impaired IFN-γ production, and depressed T-cell proliferation.[70] These defects are all reversed by the administration of exogenous prolactin.[70] Other pituitary hormones with immunoregulatory activity include follicle-stimulating hormone, luteinizing hormone,[71] and thyroid-stimulating hormone.[72]

Melatonin secreted by the pineal gland sensitizes monocytes to lipopolysaccharide (LPS) activation and enhances IL-1[73] and IFN-γ production.[74] It inhibits tumor growth and coronary atherosclerosis, besides upregulating the immune system.[75–78] Melatonin administered *in vivo*, antagonizes the immunosuppressive effects of cortisol and prevents cortisone-induced thymic atrophy.[79] It also regulates IL-12 and nitric oxide production by primary cultures of rheumatoid synovial macrophages and the THP-1 monocytic cell line, suggesting a possible role in rheumatoid arthritis.[79] Even leptin has been implicated to have a role in immune regulation as part of the adaptation to fasting[80][Table 2].

**Table 2:** Effect of various neuroendocrine factors on immune responses/cytokine levels

α-Melanocyte-stimulating hormone	Downregulates co-stimulatory molecules such as CD86, CD40; induces proliferation of suppressor factors such as cytokine synthesis, inhibitory factor IL-10. Downregulates NF-κB; suppresses Delayed Type Hypersensitivity reactions, and inhibits IL-1, IL-2, IFN-γ production in monocytes, macrophages, and dendritic cells via inhibition of NF-κB
α-Endorphin	Inhibits immunoglobulin production
Acetylcholine	Stimulates T and NK cells and increases IFN-γ production
Angiotensin II	Enhances IFN-γ production; proliferation of splenic lymphocytes; stimulates TNF-α, TGF-β, MCP-1
β- endorphin	Inhibits T cell proliferation; enhances IFN-γ production and NK cell-mediated cytotoxicity
Catecholamines	Increase Ig production, inhibit T cells and NK cells in peripheral circulation
Calcitonin-related gene peptide	Increases T cell adhesion and stimulates IL-2, IL-4, and IL-10 production; decreases IL-12, p40, and IFN-γ
DHEAS	Enhances IFN-γ and T-cell proliferation
Histamine	Inhibits IL-12, TNF, and IFN-α and enhances IL-10 production
Inhibin	Facilitates TGF-β-mediated immunosuppression in thymocytes
Activin	Inhibits TGF-β-mediated immunosuppression in thymocytes
IGF1 and IGF2	Enhances PBMC proliferation
Macrophage inhibitory factor	Blocks glucocorticoid inhibition of T cell proliferation and cytokine production
Melatonin	Enhances IL-1, IL-2, IL-6, TNF-α, and IFN-γ production
Met encephalin	Enhances antigen-specific proliferation
Nerve growth factor	Increases B-cell proliferation, IL-6 production, IL-2R expression, and IgA synthesis
Prostaglandin E2	Inhibits IL-2 production
Serotonin	Inhibits T cell proliferation, IFN-γ-induced HLA class II expression, increases NK cytotoxicity
Somatostatin	Inhibits T-cell proliferation, IFN-γ production, Ig production
Substance P	Increases T cell proliferation and IFN-γ production, causes MHC class II upregulation by IFN-γ
VIP	Inhibits T cell proliferation and IL-12, enhances IL-5 and cAMP production

IL: Interleukin; IFN: Interferon; NF: Nuclear Factor, TNF: Tumor Necrosis Factor; TGF: Transforming Growth Factor; MCP-1: Monocyte Chemotactic Protein-1; DHEAS: Dehydroepiandrosterone sulphate; PBMC: Peripheral Blood Mononuclear Cells; MHC: Major Histocompatibility Complex; cAMP: cyclic Adenosine Monophosphate; NK: Natural Killer cell; HLA: Human leucocyte Antigen

## REGULATION OF NEUROENDOCRINE SYSTEM BY CYTOKINES

Cytokines and their receptors are expressed centrally in the hypothalamus and anterior pituitary cells where they regulate pituitary development, cell proliferation, and hormone secretion. They behave as immunoneuroendocrine modulators, transducing signals that interface stress (peripheral and central) and inflammation with the hypothalamo-pituitary axes.[81] The first cytokine shown to have neuroendocrine effects was the interferon, the administration of which increases steroidogenesis. Subsequently, IL-1, IL-2 or IL-6, IFN-β, IFN-γ, LIF, and TNF-α have been shown to elevate plasma ACTH and glucocorticoid levels in both laboratory animals and humans.[82–85] IL-1, IL-2, IL-6, and TNF-α all directly stimulate the pituitary cells to produce ACTH and β-endorphins.[86] IFN-γ upregulates glucocorticoid receptor expression in macrophages during immune system activation,[87] demonstrating the action of glucocorticoid on immune cells.

By stimulating the HPA axis, cytokines also antagonize their own peripheral proinflammatory action.[88] Excessive HPA axis stimulation leads to immunosuppression and consequently to an increased susceptibility to infection, therefore, negative regulation of pituitary cytokine function is critical. Several regulators have been characterized that act at different levels of the cytokine-induced JAK2-STAT3-POMC-ACTH cascade.[89] Suppressor of cytokine signaling 3 (SOCS-3), one of the inhibitors of cytokine action[90] is potently induced in the pituitary gland.[91]

Cytokines also regulate the secretion of nonHPA axis hormones. For example, IFN-γ, granulocyte CSF, and GM-CSF stimulate melatonin secretion by the pineal gland.[87]

## EFFECT OF ACUTE AND CHRONIC STRESS ON IMMUNE SYSTEM

Two key concepts underlie the impact of stress on immune function. Firstly, most chronic, maladaptive stress has a significant psychological component for the simple reason that we succumb rapidly to physical stressors, while psychological and social strain can grind on for years. Modern brain imaging techniques offer insight into the relationships between neural networks and processes such as cognition, emotion, and memory. This intimate mind / brain relationship leads to the second important concept, namely, that the brain and immune system are closely related via functional neuroendocrine-immune pathways.[92]

Immune domains affected by stress, *i.e.*, natural *vs* specific, are consistent with the duration of the stressors—acute *vs* chronic. When stressors are acute and time-limited, there is an adaptive redistribution of cells and a preparation of the natural immune system for possible infection, injury, or both. The efficiency of this adaptive process is increased if excess energy from fight-or-flight behavior is not diverted towards these stress-related immune changes. Thus, acute stress upregulates parameters of natural immunity, which requires only a minimal time and energy investment to act against invaders.[93] In fact, energy may be directed away from the specific immune response, as indexed by the decrease in the proliferative response. The effect of brief stressors such as examinations, changes the potency of different arms of specific immunity—specifically, to shift away from cellular (Th1) immunity towards increased humoral (Th2) immunity.

Exposure to an acute laboratory stressor at the time of keyhole limpet hemocyanin (KLH) immunization in rats, results in a long-term suppression in circulating anti-KLH antibody levels, indicating that stress suppresses antigen-specific T-cells.[94]

The potential adaptiveness of the immune system declines when the stressors become more chronic. Potentially adaptive changes give way to potentially detrimental changes, initially in cellular immunity and then, more broadly in immune function. Compared with the natural immune system, the specific immune system is activated in chronic stress. As the specific immune system is time- and energy-intensive, it is invoked only when circumstances (either a stressor or an infection) persist for a longer period of time. An appropriate stress response is initiated by a stressor, sustained for an appropriate time interval, and then switched off, allowing for a recovery period. Repeated stressors may result in a lack of adaptation, prolonged responses with no recovery period, or inadequate responses.[95]

## INFLUENCE OF YOGA ON STRESS RELATED CHANGES IN IMMUNE SYSTEM

The ability to proactively handle stress in everyday life could alleviate the constant activation of the endocrine system, which in turn, increases the effectiveness of the immune system.[96] Psychoimmunological studies have used a number of diverse strategies including hypnosis, relaxation, exercise, classic conditioning, self disclosure, and exposure to phobic stressor to enhance perceived coping and self efficacy. Cognitive behavioral therapies have also been tried with different population samples and these interventions have generally produced positive changes. In bygone times, the people of ancient China and India developed methods of exercise with exceptional healing effects. Although they evolved continents apart, Qigong, t'ai chi, and yoga have certain similarities. They can all be described as meditative exercises and all involve relaxation and concentration, a focus on the breath, and gradual and purposeful movement.

Yoga is an ancient Indian culture for physical, mental, and spiritual development. The word “*yoga*” is derived from the Sanskrit root, “*yuj*”, meaning to bind, join, and yoke. This reflection of the union of the body, mind, and spirit is what differentiates yoga from general exercise programs.[97] The most commonly performed practices of *Hatha Yoga* are physical postures (*asanas*), breathing exercises (*pranayama*), and meditation (*dhyana*). *Asanas* are physical postures which stretch and strengthen different parts of the body, massaging and bringing fresh blood to the internal organs while rejevunating the nervous system and lubricating the joints, muscles, and ligaments. Each *asana* is purported to have different effects. Some are stimulatory to the nervous and circulatory systems, some develop coordination and concentration, while others have a calming effect on the body. Some postures such as the corpse pose, are used for elongated periods of relaxation. *Pranayama* consists of a variety of techniques for the regulation of breathing, usually by encouraging it to become slower, more regular, and more refined.[98] The ultimate aim of yoga is to prepare the body to achieve tranquility of the mind. Yoga has been recommended and studied in its relationship to stress, although the studies are scientifically less replicable. It has been shown to create a sense of well being, feeling of relaxation, improved concentration, self confidence, improved efficiency, good interpersonal relationships, increased attentiveness, lowered irritability, and an optimistic outlook in life.[99] Nonetheless, several researchers claim highly beneficial results from the practice of Yoga in alleviating stress and its effects. Because it fosters self-awareness, Yoga is a promising approach for dealing with the stress response. Even the western world has now accepted Yoga as a complementary therapy to assist cancer survivors in managing symptoms such as depression, anxiety, insomnia, pain, and fatigue.[97,100]

Yoga poses that twist and compress organs, help massage and rejuvenate immune organs and channels. The practice of Yoga also generates balanced energy—vital energy required by the immune system. Other key poses can create specific benefits to improve immune function:

- *Kurmasana* (tortoise pose) supports the thymus gland.
- Inversions and forward bends, *e.g*., *Adho Mukha Svanasana* (Downward Facing Dog pose) improve the flow of the sinuses and help flush mucus from the lungs.
- Chest and lung openers, *e.g*., *Ustrasana* (Camel pose), *Yoga Mudra,* and *Bhujangasana* (Camel pose) also increase lung mobility and flush out the lungs.
- Restorative Yoga poses (supported and gravity-based) can provide healing benefits during low periods of energy.

Yoga leads to an inhibition of the posterior or sympathetic area of the hypothalamus,[101] thus, optimizing the body's sympathetic responses to stressful stimuli, and restores autonomic regulatory reflex mechanisms associated with stress. Activity of the parasympathetic system may increase or remain unaffected.[99,102–104] It is also well known that the hypothalamus and the limbic system are intimately concerned with emotional expressions. Yogic practices inhibit the areas responsible for fear, aggressiveness, and rage, and stimulate the rewarding centers in the median forebrain and other areas, leading to a state of bliss and pleasure. This results in lower anxiety, heart rate, respiratory rate, blood pressure, and cardiac output in students practicing Yoga and meditation than in controls.[101,105,106]

Yogic practices probably inhibit the activity of the paraventricular nuclei of the hypothalamus, which in turn affects the anterior pituitary gland to produce less ACTH. The decrease in ACTH decreases the synthesis of cortisol from the adrenal glands. The decrease in cortisol levels with yoga has been observed in various studies.[107,108] Qi Gong training, a Chinese energy system that combines meditative techniques with other practices, was associated with an elevation in CD4 T cells and a higher CD4/CD8 cell ratio in a group of healthy practitioners than in healthy controls.[109] Qi Gong training also tends to decrease anxiety and plasma levels of ACTH, cortisol, and aldosterone.[110] Cortisol also tends to activate phenylethanolamine-*N* -methyl transferase (PNMT).[111] Consequent to sympathetic inhibition and a decrease in PNMT, catecholamine formation decreases. The decreased levels of corticosteroids and catecholamines are known to decrease stress responses.

Although not many scientifically planned clinical trials have been carried out to evaluate the effect of Yoga on various components of the immune system *per se*, many studies involving yogic practices and similar techniques have shown favorable results in immune-system mediated disorders. Studies have shown that HIV-positive men assigned to a ten-week, group-based cognitive behavioral stress management (CBSM) intervention, demonstrated positive effects on mood. These mood changes may mediate adrenal hormone regulation indicated by reductions in 24-h urinary cortisol (with reduced depressed mood) and norepinephrine (with reduced anxiety) levels and increases in serum Dehydroepiandrosterone-sulphate (DHEA-S) and testosterone levels (with reduced depressed mood). Results also suggest that CBSM-related changes in the production of these hormones may explain, in part, the effects of this intervention on the short-term changes in IgG antibody titers to herpes viruses (with increased DHEA-S-to-cortisol ratio). Also explained are longer-term changes in lymphocyte subpopulations, such as CD8 suppressor/cytotoxic cells (with reductions in urinary noradrenaline output) and transitional naïve CD4 cells (with reductions in urinary cortisol output).[112] Further evaluation in these study groups showed more CD4+CD45RA+CD29+ lymphocytes, an indicator of immune system reconstitution, at a six- to 12-months' follow-up compared with controls. Greater reductions in cortisol output and depressed mood during CBSM appeared to mediate the effects of this intervention on this indicator of immune system reconstitution over the six- to 12-months' follow-up period.[113] The probable mechanisms involved may include modulation of the HPA, HPG, and SAM axes with consequent effects on the immune system.

Meditation practices have been shown to increase melatonin levels[114] and decrease serotonin levels.[115] A statistically significant relationship has been observed between mind-body therapies (Transcendental meditation and visual imagery without the use of any kind of medications) and the patient's recovery from dermatomyositis, which could possibly mediated by influences on the humoral immune system.[116]

Many studies have shown that cancer patients have compromised immune function,[117] and immune factors have been used to predict disease progression. Levy *et al.*[118] found that less distress on the Profile of Mood States predicted a longer disease-free interval in breast cancer patients, but lower NK cell activity predicted recurrence. In a group of breast cancer patients who had recently undergone surgery, stress levels significantly predicted i) lower NK cell lysis, ii) diminished response of NK cells to recombinant IFN-γ, and iii) decreased proliferative response of peripheral blood lymphocytes to plant lectins and to a monoclonal antibody directed against the T cell receptor.[119] Sudarshan Kriya (SK) and Pranayam (*P*) are known to reduce stress and improve immune functions. A study of cancer patients who had completed their standard therapy, revealed that SK and P increased NK cells significantly (*P* < 0.001) at 12 and 24 weeks of the practice compared to baseline. Increase in NK cells at 24 weeks was significant (*P* < 0.05) compared to the controls. There was no effect on T-cell subsets after SK and P, either in the study group or among controls.[120] Fawzy *et al.*[121] provided a six-weeks' psychosocial intervention to melanoma patients and found significant increases in i) the percentage of lymphocytes and NK cells, ii) indications of increases in NK cytotoxic activity, and iii) a small decrease in the percentage of CD4 (helper) T cells. These changes were not observed immediately after the intervention but were evident after a six-month follow-up assessment.

Another pre-post intervention study showed that an eight-week mindfulness-based stress reduction program was effective in decreasing symptoms of stress and improving the overall quality of life in a group of breast and prostate cancer patients. Stress symptoms improved on eight of the ten symptoms of stress subscales, including anxiety and depression, emphasizing the breadth of stress-related symptoms that were significantly alleviated over the course of this intervention. Also, postintervention sleep quality was seen to improve in most patients. In terms of the immune results, no changes were seen in the numbers of any of the lymphocyte subtypes (NK, T, or B cells), or overall lymphocyte numbers. Postintervention decreases in IFN-γ levels, IL-10 levels (both associated with cancer-associated depression) and a 3–4 fold increase in IL-4 levels (which contributes to growth inhibition and apoptosis in breast cancer cell lines) were observed.[122]

Although it is not yet clear as to what extent these positive immunological changes translate into concrete improvements in relevant aspects of health (alterations in the incidence, severity, or duration of infectious or malignant disease), the preliminary evidence is promising.[123] Authors thus conclude that stress tends to have a negative impact on the immune system and makes a person more vulnerable to diseases. Managing stress, especially chronic or long-term stress (even if it is not intense), by practising various relaxation techniques, may help people overcome other co-morbidities associated with diseases and lead a better quality of life even during periods of stress.
